# Targeted mutation of NOV/CCN3 in mice disrupts joint homeostasis and causes osteoarthritis-like disease

**DOI:** 10.1016/j.joca.2014.12.012

**Published:** 2015-04

**Authors:** K.A. Roddy, C.A. Boulter

**Affiliations:** School of Biosciences, Cardiff University, Museum Avenue, Cardiff CF10 3AX, UK

**Keywords:** NOV/CCN3, Joint homeostasis, Osteoarthritis, Targeted mouse mutant

## Abstract

**Objective:**

The matricellular protein NOV/CCN3, is implicated in osteoarthritis (OA) and targeted mutation of NOV in mice (*Nov*^*del3*^) leads to joint abnormalities. This investigation tested whether NOV is required for joint homeostasis and if its disruption causes joint degeneration.

**Method:**

NOV expression in the adult mouse joint was characterized by immunohistochemistry. A detailed comparison of the joints of *Nov*^*del3*^−/− and *Nov*^*del3*^+/+ (wild-type) males and females at 2, 6 and 12 months of age was determined by X-ray, histology and immunohistochemistry.

**Results:**

NOV protein was found in specific cells in articular cartilage, meniscus, synovium and ligament attachment sites in adult knees. *Nov*^*del3*^−/− males exhibited severe OA-like pathology at 12 months (OARSI score 5.0 ± 0.5, *P* < 0.001), affecting all tissues of the joint: erosion of the articular cartilage, meniscal enlargement, osteophytic outgrowths, ligament degeneration and expansion of fibrocartilage. Subchondral sclerosis and changes in extracellular matrix composition consistent with OA, were also seen. The density of articular cartilage cells in *Nov*^*del3*^+/+ knee joints is maintained at a constant level from 2 to 12 months of age whereas this is not the case in *Nov*^*del3*^−/− mice. Compared with age and sex-matched *Nov*^*del3*^+/+ mice, a significant increase in articular cartilage density was seen in *Nov*^*del3*^−/− males at 2 months, whereas a significant decrease was seen at 6 and 12 months in both *Nov*^*del3*^−/− males and females.

**Conclusion:**

NOV is required for the maintenance of articular cartilage and for joint homeostasis, with disruption of NOV in ageing *Nov*^*del3*^−/− male mice causing OA-like disease.

## Introduction

An impaired ability to maintain homeostasis of the joint is associated with tissue degeneration, yet the mechanisms that regulate this process are largely unknown. Failure to maintain healthy articular cartilage cells is a common feature of ageing and an important underlying cause of osteoarthritis (OA)^1^. The association of OA with impaired joint homeostasis suggests common regulators in the pathways mediating these processes.

NOV (CCN3) is a member of the CCN family of matricellular proteins which have important roles in development, wound healing, angiogenesis and disease. In skeletal development and disease CTGF (CCN2) is required for coordinating chondrogenesis and angiogenesis^2^, WISP1 (CCN4) is upregulated in OA^3^ and mutations in WISP3 (CCN6) cause progressive pseudorheumatoid dysplasia^4^, while NOV (CCN3) has roles in skeletal development^5^, regulation of osteogenesis^6,7^, bone regeneration^8^ and bone cancer^9^. All CCN proteins share a common modular structure: an insulin-like growth factor binding protein (IGFBP) domain, a von Willibrand factor type C (WVC) domain, a thrombospondin type 1 repeat (TSP1) and a carboxy-terminal (CT) domain containing a cysteine knot (absent in CCN5). By specific interactions with these domains, CCN proteins modulate multiple signalling pathways including BMPs, Wnt, TGFs, Notch and integrins to regulate cell proliferation, survival, adhesion, migration and differentiation^10^.

Recent work has implicated NOV in OA. In experimentally induced OA in mice elevated NOV expression is seen in gene array studies using RNA isolated from arthritic joints^11^. Similarly, in human OA NOV is upregulated^12^. This may reflect a role for NOV in OA pathology or alternatively, that NOV is induced in response to joint damage and acts to mediate repair. Indeed, studies comparing OA susceptible and non-susceptible rat strains have identified NOV as a potential gene conferring protection from OA^13^.

Our preliminary characterization of mice homozygous for the targeted mutation *Nov*^*del3*^ revealed joint abnormalities in addition to other mild skeletal phenotypes^5^. In this paper, we have performed a detailed investigation of the role of NOV in the adult joint. We have characterized the expression pattern of NOV in the adult knee joint, and show that homeostasis of articular cartilage cells is compromised in the *Nov*^*del3*^−/− mutants, and that 12 month *Nov*^*del3*^−/− males exhibit joint degeneration with an OA-like pathology.

## Materials and methods

### Experimental animals

Male and female *Nov*^*del3*^−/− mice carrying a targeted mutation in Nov^5^ and wild type littermates (*Nov*^*del3*^+/+) were maintained on a 129Sv background. Breeding and analysis of mice were approved by the Cardiff University's Ethical Committee and performed in accordance with the Animal [Scientific Procedures] Act 1986 under project licences PPL 30/2672, PPL 30/2600. Mice housed together on a 12-h light–dark cycle with a temperature range of 21C ± 2, with free access to tap water and standard chow.

### X-ray imaging and skeletal preparation

*Nov*^*del3*^+/+ and *Nov*^*del3*^−/− male and female adult mice were fixed by perfusion with 4% paraformaldehyde (PFA) in PBS at 2, 6 and 12 months (*n* = 6 for each sex and genotype at each time point). The hind limbs were dissected and imaged by X-ray (KODAK *In-Vivo* Imaging System FX Pro) prior to decalcification in 10% EDTA pH 7. 7 μm coronal paraffin sections were prepared semi-serially.

### Histological assessment and OARSI score

Comparable sections from each animal, stained using haematoxylin and eosin (H&E), toludine blue and safranin O were assessed for histopathological changes (by KR blinded to genotype), using the OARSI scheme^14^ (see Supplementary Methods), assigning a score between 0 and 6 to all four quadrants averaged over three sections (*n* = 6 of each sex and genotype at each time point). OA severity is expressed as the summed score across the entire joint. Collagen birefringence in the tibial plateau (two sections, *n* = 4 for each sex and genotype at each time point) was imaged by polarized light microscopy (Polarisation equipped Zeiss Photomicroscope).

### Analysis of articular cartilage cell density and thickness

Articular cartilage cell density was determined for each of the four quadrants of the joint from four H&E sections per animal (*n* = 4 for each sex and genotype at each time point) (KR blinded to genotype). Cell number and area of articular and calcified cartilage were measured within a box of 0.32 × 0.23 mm placed on load-bearing regions of articular cartilage. Cartilage thickness, measured at three equidistant points across the cartilage on both the medial and lateral side of the tibia, was averaged across a minimum of three sections (*n* = 4 for each sex and genotype at each time point).

### Immunohistochemistry

Anti-NOV rabbit polyclonal antibody Novpep5170 (Pepceuticals Ltd), against peptide CPQNNEAFLQDLELKTSRGEI, was used to analyse expression in 6 month *Nov*^*del3*^+/+ and *Nov*^*del3*^−/− males and females (*n* = 3 for each genotype and sex). Cell proliferation and apoptosis were analysed at 2, 6 and 12 months using a rabbit polyclonal to PCNA (Abcam) and PARP p85 (Promega), (*n* = 4 for each genotype, sex and stage). The percentage positive cells was determined for each of four quadrants, averaged over two slides by KR blinded to genotype. Collagen I and Collagen X expression was assayed using mouse monoclonals anti-ColI (1/1000, Sigma) and anti-ColX (1/10, gift of Klaus von der Mark) (*n* = 4 for each genotype, sex and stage). Immunohistochemistry details in Supplementary Methods.

### Statistical analysis

The effects of genotype, sex and age on measurements of articular cartilage histological (OARSI) score, thickness and cell density, and chondroctye proliferation/apoptosis were tested using three way ANOVA (SPSS) and included analysis of both simple and main effects. Normality was checked using the Shapiro–Wilk test and homogeneity of variances was verified using Levene's test (SPSS).

## Results

### NOV is expressed in the adult knee joint

**Fig. 1:**
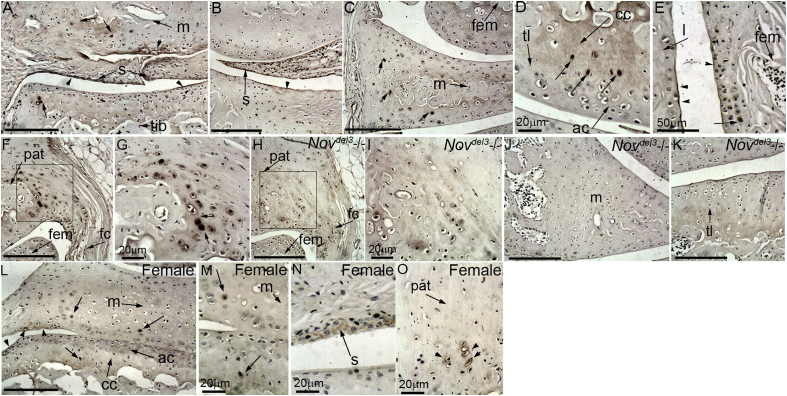
Immunohistochemical characterization of NOV expression in 6 month males (A–K) and females (L–O). NOV is expressed in the most superficial layer of the articular cartilage at margins of the joint and meniscus (A, B, arrowhead), the synovial intima (A, B) and a subset of cells in the calcified layer (A, D, arrow) and meniscus (B). NOV is also present in the synovial lining of the femur and lining the collateral ligaments (E), and in the attachment site of the fibrous capsule (fc) to the patella (F, G). No signal was detected in *Nov*^*del3*^−/− fibrous capsule (H, I), meniscus (J) or articular cartilage (K). In females NOV is also expressed in the superficial layer (L, arrowhead), the synovial intima (N), a subset of cells in the calcified layer and meniscus (L, M) and the patella (O). Scale bars are 100 μm unless indicated. tib; tibia, m; meniscus, s; synovium, fem; femur, ac; articular cartilage, tl; tideline, cc; calcified cartilage, l; collateral ligament, pat; patella.

NOV was detected by immunohistochemistry in multiple tissues of male and female knee joints: the synovial intima, a membrane lining all the non-articular structures in the joint [Fig. 1(A), (B), (N)]; the superficial layer of the articular cartilage, particularly at the extremities of the joint e.g., near the cruciate ligament [Fig. 1(A), (B) arrowhead, L]; a subset of cells in the calcified cartilage [Fig. 1(A), (D) (arrow), L]; the outmost layer of cells in the meniscus [Fig. 1(A)] and a subset of cells in the core [Fig. 1(A), (C) (arrow), M]; the synovial lining over the femur and in a thin layer lining the inner surface of the adjacent collateral ligament [Fig. 1(E)]; the patella, in large round cells at the fibrocartilage attachment sites of the capsular ligaments [Fig. 1(F), (G), (O)]. No signal was detected in *Nov*^*del3*^−/− mice [Fig. 1(H)–(K)].

### Nov^del3^−/− mice exhibit joint defects with features characteristic of OA

**Fig. 2:**
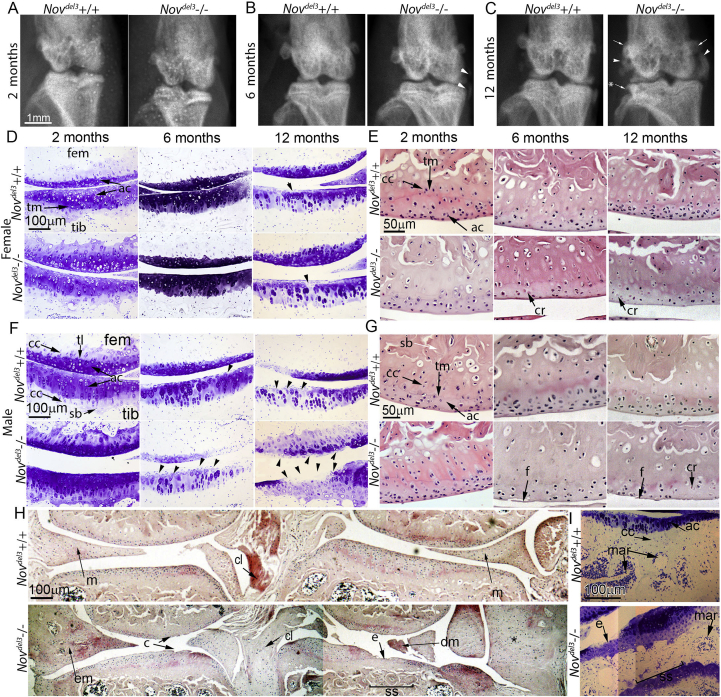
X-ray and histological analysis of *Nov*^*del3*^−/− and *Nov*^*del3*^+/+ knee joints. A–C: X-rays of 2, 6 and 12 month male *Nov*^*del3*^−/− joints revealed abnormal bone (B, C, arrowhead), osteophytes (C,*), enlarged epicondyles (C, arrow), whereas male *Nov*^*del3*^+/+ joints were normal. Knee joints of both *Nov*^*del3*^−/− and *Nov*^*del3*^+/+ females were normal (data not shown). D–G: Histological analysis of 2, 6 and 12-month-old *Nov*^*del3*^−/− and *Nov*^*del3*^+/+ females (D, E) and males (F, G) by staining with toluidine blue (D, F) and H and E (E, G). Normal toluidine blue staining of *Nov*^*del3*^−/− females compared with controls (D), but H and E staining showed decreased articular cartilage cell densities at 2, 6 and 12 months (E) with cell remnants (cr) present at 6 and 12 months (E) (see inserts). Compared to age and sex matched controls, *Nov*^*del3*^−/− male joints had reduced toluidine blue staining (F), depleted articular cartilage cell density and articular damage at 6 and 12 months (G) with fibrillation (f) at 6 and 12 months. H: H and E staining of *Nov*^*del3*^−/− and *Nov*^*del3*^+/+ males at 12 months showing significant joint pathology in the mutant with erosion (e), clefts (c), enlarged (em) or damaged (dm) meniscus (m), cruciate ligament (cl) degeneration and fibrocartilage expansion (*) in the joint margins and subchondral sclerosis (ss). I: 12 month males showed distinct subchondral sclerosis (ss) with loss of marrow cavities (mar). tib; tibia, fem; femur, ac; articular cartilage, cc; calcified cartilage, tm; tidemark. Scale bar 1 mm (A–C), 100 μm (D, F, H) and 50 μm (E, G).

To determine the effect of NOV expression on the gross anatomy of the knee joint, *Nov*^*del3*^−/− males and females were analysed by X-ray at 2, 6 and 12 months of age and compared with age and sex matched *Nov*^*del3*^+/+ littermates (*n* = 6 for each group) [Fig. 2(A)–(C)]. While the structure of the female joint at all time points appeared overtly normal (not shown), the *Nov*^*del3*^−/− males displayed a number of pathological changes compared with *Nov*^*del3*^+/+ males [Fig. 2(B), (C)]. The menisci of 12 month *Nov*^*del3*^−/− males (4/6) were abnormally large with regions of calcification adjacent to the knee [Fig. 2(C) arrowhead], as previously noted in Ref. 5. While normal at 2 months [Fig. 2(A)], early stages of meniscal enlargement could already be seen at 6 months (1/6) [Fig. 2(B) arrowhead]. At 12 months, osteophytes were seen, particularly on the tibia [Fig. 2(C)] and the femoral epicondyle also appeared enlarged [Fig. 2(C)] (4/6).

Histological analysis was performed by toluidine blue staining of cartilage extracellular matrix (ECM) and H&E staining of coronal sections of the knees isolated from *Nov*^*del3*^−/− and *Nov*^*del3*^+/+ males and females at 2, 6 months and 12 months of age (*n* = 6 for each group) (Fig. 2). *Nov*^*del3*^−/− female mice were comparable to *Nov*^*del3*^+/+ controls at 2, 6 and 12 months, with both showing slight reductions in toluidine blue staining at 12 months [Fig. 2(D) arrowhead]. The number of articular cartilage cells at 6 and 12 months appeared reduced compared to *Nov*^*del3*^+/+ females, with evidence of cell remnants near the articular surface [Fig. 2(E)]. In contrast, the joints of *Nov*^*del3*^−/− males were clearly abnormal [Fig. 2(F)–(H)]. Toluidine blue staining of cartilage ECM was increased in 2 month *Nov*^*del3*^−/− males compared with *Nov*^*del3*^+/+ controls [Fig. 2(F)], consistent with the increased numbers of articular cartilage cells also seen [Fig. 2(G)]. However, at 6 and 12 months toluidine blue staining was reduced in *Nov*^*del3*^−/− males compared to *Nov*^*del3*^+/+ controls [Fig. 2(F)], consistent with the depletion of articular cartilage cells also observed [Fig. 2(G)]. Six-month-old *Nov*^*del3*^−/− males also showed distinct fibrillation of the articular surface [Fig. 2(F), (G)] and by 12 months the male joint was badly compromised [Fig. 2(F), (H)]. Severe pathological changes included loss of ECM staining [Fig. 2(F)], cracks in the articular surface, loss of chondrocytes and loss of the articular surface, with exposure of bone and damage to the meniscus [Fig. 2(H)]. Enlarged menisci with peripheral marrow cavities were present in 12 month *Nov*^*del3*^−/− males [Fig. 2(H)]. Abnormal fibrocartilage-like tissue was also found around the knee joint in spaces normally occupied by synovium or collateral ligaments. Subchondral sclerosis was evident at 12 months in *Nov*^*del3*^−/− males, but not in *Nov*^*del3*^+/+ males [Fig. 2(I)] or in females of either genotype (data not shown). In summary, the pathological changes observed in the *Nov*^*del3*^−/− males were consistent with an OA-like phenotype.

**Fig. 3:**
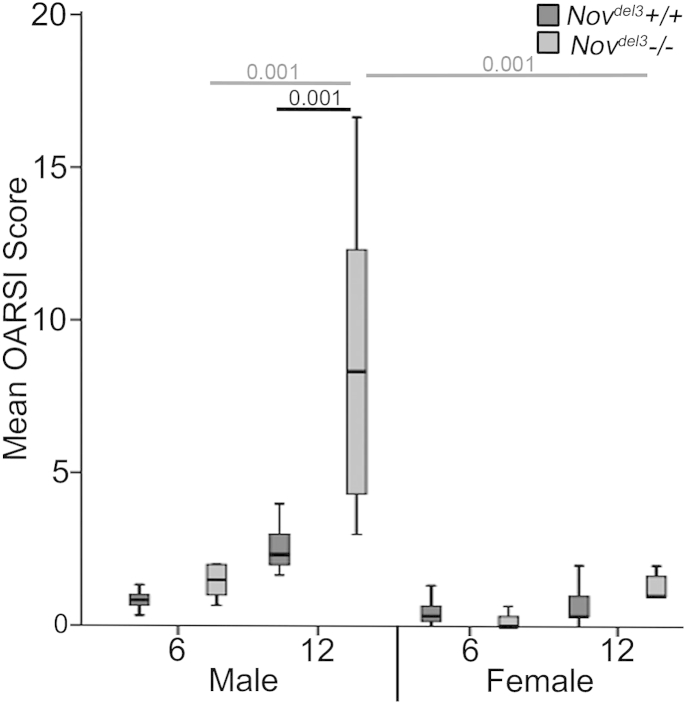
Mean OARSI score of the knee joints of *Nov*^*del3*^−/− and *Nov*^*del3*^+/+ males and females at 6 and 12 months (*n* = 6 of each sex and genotype at each time point), (±95% CI), *P* values indicated.

To determine the severity of OA in the *Nov*^*del3*^−/− mice, the OARSI grading scheme^14^ was used. No pathological changes were apparent at 2 months in *Nov*^*del3*^−/− or *Nov*^*del3*^+/+ males or females (OARSI score of 0, data not shown). No significant changes in the OARSI scores were observed between *Nov*^*del3*^−/− mice and sex-matched controls at 6 months. However, at 12 months *Nov*^*del3*^−/− males, but not females, showed significantly higher OARSI scores (Fig. 3; OARSI score 5.0 ± 0.5, *P* < 0.001), indicating severe OA-like joint pathology. The OARSI score of the 12 month *Nov*^*del3*^−/− males was also significantly higher than those of the 6 month *Nov*^*del3*^−/− males (*P* < 0.001) and 12 month *Nov*^*del3*^−/− females (*P* < 0.001). No significant differences were seen between the *Nov*^*del3*^+/+ males and females.

### Abnormal differentiation in multiple joint tissues in male *Nov*^*del3*^−/− mice

**Fig. 4:**
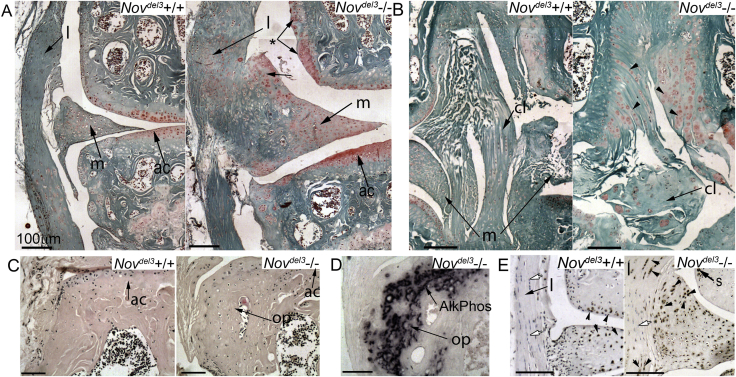
Histological changes at 12 months in knee joints of *Nov*^*del3*^−/− males compared with *Nov*^*del3*^+/+ males. A, B: safranin O staining. Increased staining seen in the meniscus (m), articular cartilage (ac), collateral ligament (l), cruciate ligament (cl) and synovium (*) (A, B). Note expansion of fibrocartilage stained with safanin O in the collateral ligament (l) (A) and cruciate ligament (cl) (arrowheads) (B). C: H and E staining of joint margins showing the presence of osteophytic outgrowths (op) in *Nov*^*del3*^−/− males (C), but not in *Nov*^*del3*^+/+ control. D: Alkaline phosphatase staining of adjacent section shown in C. Positive staining of the osteophytes with the osteoblast marker alakaline phosphatase (AlkPhos) (D). E: Immunohistochemistry with anti-PCNA antibody, counterstained with haematoxlyin. Proliferating cells expressing PCNA (black arrowhead) were found in ligaments (l), synovium (s) and in the groups of cells in the margins of the meniscus in *Nov*^*del3*^−/− males. Negative cells indicated by white arrow. Scale bar 100 μm.

Safranin O and toluidine blue staining revealed that proteoglycans characteristic of cartilage were present in multiple ectopic sites in the joints of 12 month *Nov*^*del3*^−/− males, indicating inappropriate cartilage differentiation in these tissues, but not in *Nov*^*del3*^−/− females and *Nov*^*del3*^+/+ control mice of either sex. Increased safranin O staining was found at multiple sites in the enlarged menisci of *Nov*^*del3*^−/− males, particularly at the periphery [Fig. 4(A)], in the synovial intima lining the femur [Fig. 4(A),*], in the collateral ligament and throughout the femoral notch [Fig. 4(B)], an area normally occupied by the cruciate ligaments. In the ligaments, proteoglycan changes ranged from abnormal safranin O staining near the insertion sites to complete degeneration of the cruciate ligament and its replacement with extensive safranin O stained fibre-like structures [Fig. 4(B)], possibly indicating cartilage metaplasia in the ligament. Osteophytes, expressing the osteoblast marker alkaline phosphatase, were also found in *Nov*^*del3*^−/− male mice at 12 months [Fig. 4(C), (D)] and in males at 6 months, but not in *Nov*^*del3*^+/+ males or in females of either genotype (data not shown). Abnormal clusters of proliferating cells were present in the meniscus of *Nov*^*del3*^−/− males at 12 months but not in *Nov*^*del3*^+/+ male littermates, based on immunohistochemistry with an anti-PCNA antibody [Fig. 4(E)]. Increased numbers of PCNA positive cells were also identified in the synovial intima over the femur and in the collateral ligaments [Fig. 4(E)] and cruciate ligament (not shown) of *Nov*^*del3*^−/− males. This suggests that NOV mutation is associated with ectopic cell proliferation in joint tissues with an OA-like pathology.

### Articular cartilage cell density and thickness is altered in *Nov*^*del3*^−/− mice

**Fig. 5:**
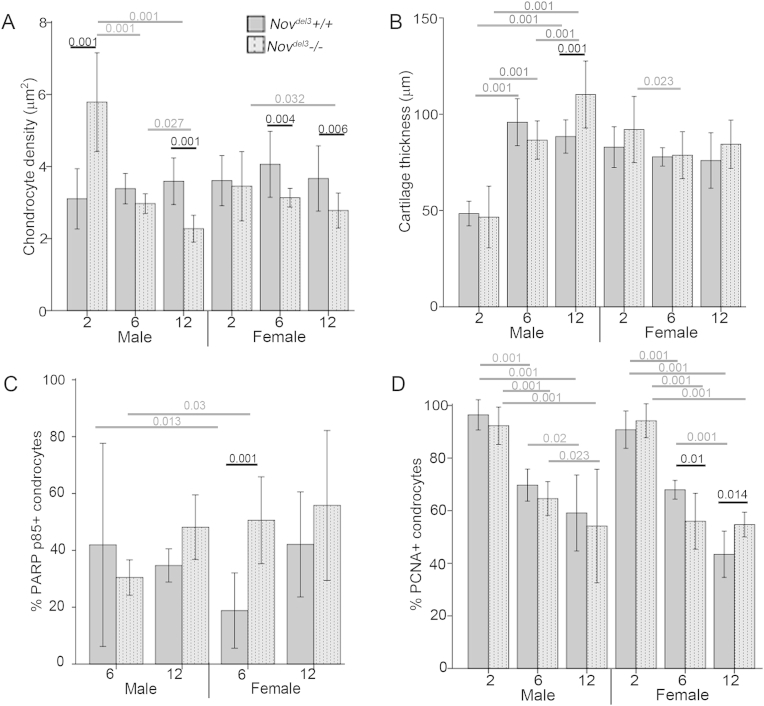
Histological analysis of articular chondrocytes in *Nov*^*del3*^−/− and *Nov*^*del3*^+/+ knee joints at 2, 6 and 12 months. A: Chondrocyte density measurements (*n* = 4 at each age, genotype and sex). B: Cartilage thickness (*n* = 4 at each age, genotype and sex). C: Percentage of cells positive for immunohistochemical staining with anti-PARP p85 antibody apoptotic marker (*n* = 4 at each age, genotype and sex). D: Percentage of cells positive for immunohistochemical staining with anti-PCNA antibody proliferation marker (*n* = 4 at each age, genotype and sex). No PARP p85 positive cells were detected at 2 months (data not shown). Graphs depict mean (±95% CI), *P* values indicated.

Initial observations of H&E stained sections indicated differences in the density of articular cartilage cells in *Nov*^*del3*^−/− mice [Fig. 2(E), (G)]. To quantify this, the mean density of articular chondrocytes was determined for the medial and lateral compartments of the femur and tibia at each time point and the overall mean used for analysis (*n* = 4 mice of each genotype and sex at each time point). Articular cartilage cell density is maintained at a constant level in *Nov*^*del3*^+/+ mice over the timescale 2–12 months [Fig. 5(A)]. In contrast, this is not seen in *Nov*^*del3*^−/− mice. Compared to aged matched *Nov*^*del3*^+/+ males, a significant increase in articular cartilage cell density was found in *Nov*^*del3*^−/−males at 2 months (*P* < 0.001) while a significant decrease was seen at 12 months (*P* < 0.001) [Fig. 5(A)]. Articular cartilage cell densities were also significantly reduced in *Nov*^*del3*^−/− females at 6 months (*P* = 0.004) and 12 months (*P* = 0.006) compared with age matched *Nov*^*del3*^+/+ females [Fig. 5(A)].

The mean thickness of the articular cartilage was also measured, in order to determine whether this might account for the alterations in cell density [Fig. 5(B)]. In 12-month-old *Nov*^*del3*^−/− males, a significant increase in articular cartilage thickness was observed (*P* < 0.001). However, compared to sex and aged matched controls, no significant differences were observed in *Nov*^*del3*^−/− females at any timepoint and in *Nov*^*del3*^−/− males at 2 and 6 months, indicating that other factors contributed to the early cell density changes seen in these mice.

**Supplementary Fig. 1:**
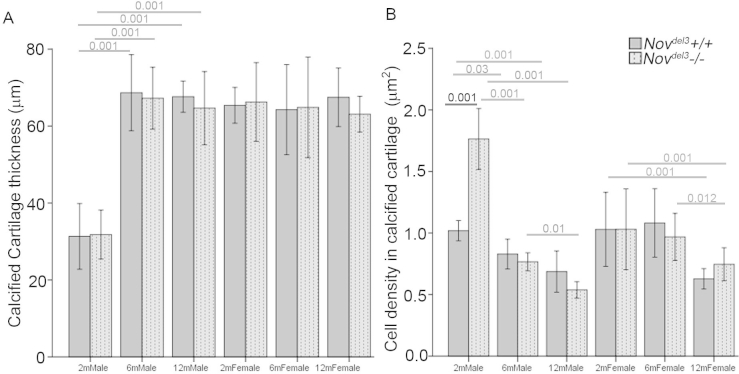
Histological analysis of calcified cartilage in *Nov*^*del3*^−/− and *Nov*^*del3*^+/+ knee joints at 2, 6 and 12 months. A: Calcified chondrocyte density measurements (*n* = 4 at each age, genotype and sex). B: Calcified cartilage thickness (*n* = 4 at each age, genotype and sex). Graphs depict mean (±95% CI), *P* values indicated.

In contrast to the articular cartilage, the calcified cartilage was much less severely affected, with no significant changes to the overall thickness, comparing *Nov*^*del3*^−/− and *Nov*^*del3*^+/+ age and sex-matched mice at 2, 6 and 12 months [Supplementary Fig. 1(A)]. Interestingly, at 2 months calcified cartilage was significantly thinner in males of both genotypes than in females and older males (*P* < 0.001). Similarly, no significant changes in chondrocyte cell density were seen in the calcified cartilage of *Nov*^*del3*^−/− and *Nov*^*del3*^+/+ sex matched mice at 6 and 12 months [Supplementary Fig. 1(B)], although a significant increase was noted in 2 month *Nov*^*del3*^−/−males (*P* < 0.001). Thus, the calcified cartilage appears less severely affected by *Nov* mutation than is the articular cartilage.

### Altered cell proliferation and cell death in Nov^del3^−/− mice

To determine whether articular cartilage cell density changes might reflect altered apoptosis or cell proliferation, immunohistochemistry for the apoptosis marker PARP p85 [Fig. 5(C)] and the proliferation marker PCNA [Fig. 5(D)] was performed. A significant increase in the percentage of PARP p85 and significant decrease in the percentage of PCNA positive cells were observed in 6-month-old females (*P* < 0.001 and *P* = 0.01, respectively), indicating that increased apoptosis and decreased proliferation contribute to the decrease in articular cartilage cell density of *Nov*^*del3*^−/− females at this time point [Fig. 5(C), (D)]. However, this was not observed in 12-month-old *Nov*^*del3*^−/− females where no significant difference was seen in the percentage of apoptotic cells and a significant increase (*P* < 0.014) in proliferation was apparent compared to control mice, despite a decrease in articular cartilage cell density. No significant differences were seen in the percentage of PCNA or PARP p85 positive cells in 2, 6 and 12 month *Nov*^*del3*^−/− males compared to *Nov*^*del3*^+/+ males, indicating that altered rates of cell proliferation or apoptosis could not account for the cell density differences observed in these mice [Fig. 5(C), (D)].

### Abnormal ECM in *Nov*^*del3*^−/− mice characteristic of OA

**Fig. 6:**
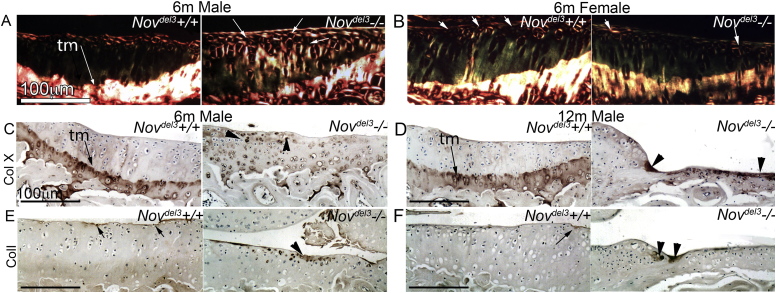
Collagen content and distribution in articular cartilage of knee joints of *Nov*^*del3*^−/− mice and *Nov*^*del3*^+/+ mice. A, B: Collagen birefringence, captured from picrosirus Red stained sections of 6 month males (A) and females (B) imaged under polarized light. Thicker collagen fibres seen in *Nov*^*del3*^−/− males compared with *Nov*^*del3*^+/+ males (A, white arrows) but not in *Nov*^*del3*^−/− females (B). C–F: Immunohistochemistry with anti-collagen X (C, D) and anti-collagen I antibodies (E, F) of *Nov*^*del3*^−/− and *Nov*^*del3*^+/+ male articular cartilage at 6 (D, E) and 12 months (D, F). In *Nov*^*del3*^+/+, collagen X is adjacent to the tidemark (tm) (A, B, arrows) and collagen I is in the superficial articular cartilage (C, D, arrows). Aberrant expression of collagen I and collagen X in *Nov*^*del3*^−/− males at 6 and 12 months is indicated by arrowheads (C–F). Scale bar 100 μm.

Specific changes to the distribution of ECM molecules in articular cartilage are indicative of OA, including altered collagen fibre structure^15^ and aberrant expression of collagen I and collagen X^15^. We determined the structure of collagen in articular cartilage of 6 month *Nov*^*del3*^−/− and *Nov*^*del3*^+/+ mice by picrosirus red staining and polarised light microscopy (*n* = 4 for each sex and genotype). Brighter and strikingly thicker collagen fibril structure was seen in *Nov*^*del3*^−/− males compared with *Nov*^*del3*^+/+ males [Fig. 6(A)]. In contrast, no obvious structural changes were apparent in *Nov*^*del3*^−/− females compared with controls [Fig. 6(B)].

Collagen X is a marker of hypertrophy in chondrocytes and is normally restricted in the joint to the thin layer of calcified cartilage below the tidemark. However in OA, collagen X is expressed in cells above the tidemark^16,17^, consistent with abnormal differentiation of articular cartilage cells down the endochondral pathway. Immunohistochemistry using an anti-collagen X antibody gave the expected pattern of expression in knee joints in *Nov*^*del3*^+/+ males and females at 6 and 12 months [Fig. 6(C), (D); data not shown]. *Nov*^*del3*^−/− females at 6 and 12 months also expressed collagen X as normal (data not shown). In contrast, in joints of *Nov*^*del3*^−/− males, the normal expression of collagen X at the tidemark was disrupted [Fig. 6(C), (D)] and was also present near areas of obvious cartilage damage [Fig. 6(C), (D)], consistent with an OA-like pathology.

Collagen I is normally expressed in the superficial layer of the articular cartilage and its expression increases in areas of damage^17–19^. As expected, *Nov*^*del3*^+/+ males at 6 and 12 months showed normal collagen I localization [Fig. 6(E), (F)]. In contrast, 6 and 12 month *Nov*^*del3*^−/− males exhibited stronger collagen I staining in the articular cartilage surface layer and in areas of visible cartilage damage [Fig. 6(F)].

## Discussion

This is the first detailed characterization of the joint phenotype in *Nov*^*del3*^ mutant mice and identifies NOV as a new regulator of joint homeostasis with a role in OA. Homeostasis of articular cartilage is crucial to joint health. Here we show that the density of articular cartilage cells is normally maintained at a constant level in adult 129Sv mice between 2 and 12 months of age, whereas, this homeostatic process is compromised in *Nov*^*del3*^−/− mutants. *Nov*^*del3*^−/− females exhibited depletion of articular cartilage cell density compared to age matched *Nov*^*del3*^+/+ females at 6 and 12 months; at 6 months this was due, at least in part, to decreased proliferation and increased apoptosis. In contrast, altered proliferation and apoptosis could not account for the large variation seen in articular cartilage cell densities in *Nov*^*del3*^−/− males where, compared with age-matched *Nov*^*del3*^+/+ males, a significant depletion in cell density was seen at 12 months, whereas a dramatic increase was observed at 2 months. This suggests that differences in the density of articular chondrocytes in *Nov*^*del3*^−/− males have a different underlying origin, possibly reflecting changes in development or in behaviour of articular cartilage cells or their progenitor populations, as discussed below. Thus, normal NOV function is essential for maintenance of articular cartilage cell density in adult mice in the first year of life.

Multiple lines of evidence indicate loss of normal NOV function in *Nov*^*del3*^−/− males leads to OA-like joint pathology. Moderate pathological changes are present in 6 month *Nov*^*del3*^−/− males and, by 12 months, severe joint pathology is seen, reflected in a high OARSI score. Subchondral bone is less severely affected with subchondral sclerosis, but little effect on the calcified cartilage. Collagen X positive cells near damaged articular surfaces indicate aberrant differentiation of articular chondrocytes, also seen in OA^16^. In *Nov*^*del3*^−/− mice, clusters of cells in the margins of the meniscus and in the synovium express the proliferation marker PCNA, consistent with the expansion of these tissues. Severe disruption of the knee joint is apparent at 12 months in *Nov*^*del3*^−/− males with osteophytes, degenerate ligaments, enlarged menisci and abnormal proteoglycan content. The worst cruciate ligament degeneration is associated with the presence of the largest menisci and most osteophytes. Abnormal ligaments and menisci would significantly alter the distribution of load across the joint, leading to changes in gene expression in the mechanically sensitive cartilage and ligaments. Abnormal loading of the joint is a key factor in the progression and severity of OA^20–22^ and is likely to be a contributing factor in the early onset OA-like pathology seen in the *Nov*^*del3*^−/− mice. Moreover, joint laxity has also been observed in *Nov*^*del3*^−/− mice, correlating with the expression of NOV in mechano-responsive ligament attachment sites and tendons^5^. The meniscus, important in correct load transmission and abnormal in *Nov*^*del3*^−/− males, is also a site of NOV expression. Indeed, NOV has been shown to be regulated by mechanical stress^23^. The expression of NOV in a subset of cells in joint tissues may reflect several parameters including differentiation, cell cycle status or mechanical load. Thus, the aberrant behaviour of joint tissues in *Nov*^*del3*^−/− mice may result directly from NOV mutation, rather than being solely a consequence of abnormal joint structure and biomechanics. Whilst joint defects have not previously been reported in another NOV mouse mutant^7^, this may reflect differences in the mutation or strain in the proclivity of mice to develop OA-like pathology.

The ECM of the articular cartilage in *Nov*^*del3*^−/− male mice was also significantly different at 6 months, with decreased levels of proteoglycans and increased collagen fibre thickness compared to age and sex-matched littermates. Consistent with this, NOV has been reported to promote sulphated proteoglycan synthesis in chondrocytes isolated from developing rat epiphysis^24^. It would be of interest to determine whether ECM proteins that regulate collagen fibril size and diameter, such as decorin and collagen IX are altered in the *Nov*^*del3*^−/−mice. NOV has been shown to bind to fibrillin-1, and, is over expression in Tsk mice is associated with increased fibrillin-1 expression and repression of microfibril assembly^25^. Given that fibrillin-1 has recently been implicated in OA^26^, it would also be of interest to determine whether fibrillin-1 staining in the mutant is affected.

The observation that male *Nov*^*del3*^−/− mice are more affected than age-matched females is consistent with other mouse models of OA^27^. The higher incidence of spontaneous OA in male mice has been linked directly to the action of male hormones in exacerbating OA pathology^27^ and the protective role of female hormones^28^, suggesting that there may be a regulatory network involving sex hormones that influences OA severity. The androgen receptor has recently been shown to inhibit the Nov promoter *via* epigenetic silencing^29^. The increased susceptibility of *Nov*^*del3*^−/−males to OA cannot be explained through the androgen inhibition of the Nov promoter, but it may suggest that in mice NOV is part of a wider regulatory network involving sex hormones that leads directly or indirectly to increased OA susceptibility.

NOV has previously been linked to OA through its increased expression demonstrated in micro-array studies in mouse^11^ and in man^12^ suggesting a role for NOV in OA processes. We believe that these results can be reconciled with ours by the hypothesis that NOV is induced in damaged tissue in OA and plays a role in healing. Indeed, a role for NOV in tissue regeneration has been reported in the skin^30^ and tooth^31^. Articular cartilage is normally very limited in its ability to heal and it appears that this is further compromised in *Nov*^*del3*^−/− mice. Cartilage homeostasis and repair is thought to involve expansion and differentiation of progenitor cell populations; putative stem/progenitor cells are located in the synovium^32^ and in the superficial layer of the articular cartilage^33,34^ and a population of stem/progenitor cells has been shown to migrate from below the tidemark in late stage OA^35^. These cells are capable of differentiating and some have been shown respond to damage and to attempt healing^33^. Expression of NOV in the articular cartilage superficial layer and synovium suggests that it might influence the behaviour of the progenitor cells located there. Indeed, a role for NOV in regulating precursor cells has been shown in other systems^31,36,37^. It will be of interest to determine whether the alterations in articular chondrocyte density seen in *Nov*^*del3*^−/− mice is due to abnormal progenitor cell function; abnormal stem cell activation in young mice, could result in subsequent stem cell depletion, thereby contributing to the decreased articular cartilage cell density in older mice. NOV is also reported to direct articular chondrocyte differentiation and inhibit endochondral differentiation^38,39^. Our data is consistent with this: inappropriate collagen X staining is seen in *Nov*^*del3*^−/− males at 6 months, prior to serious OA-like pathology.

The *Nov*^*del3*^−/− mutant is a new mouse model for OA which crucially affects multiple tissues of the joint and arises during ageing, with many features seen in human OA. Importantly, unlike many other animal models of OA, disease onset is not dependent on surgical intervention to destabilize the medial meniscus or damage the ligaments^40–42^, allowing the involvement of these tissues in OA progression to be studied. The *Nov*^*del3*^ mutation thus provides a new model to study joint ageing and degeneration in a known genetic background, potentially allowing better understanding of the early changes induced in OA throughout the entire structure of the joint.

## Author contributions

KAR co-designed, performed and analysed the experiments, and co-wrote manuscript. CAB obtained funding, co-designed and supervised project and co-wrote manuscript.

## Role of the funding source

This work was funded by a project grant to CAB from the charity Arthritis Research UK (ref. 19283).

## Conflict of interest

The authors declare no conflicts of interest.
