# Supplementary material for: Targeted mutation of NOV/CCN3 in mice disrupts joint homeostasis and causes osteoarthritis-like disease
Source: Osteoarthritis Cartilage. 2015 Apr;23(4):607–15. doi: 10.1016/j.joca.2014.12.012 (PMC4373756; doi:10.1016/j.joca.2014.12.012)
Supplement: Supplementary file 1 [file mmc1.docx]

**Supplementary Methods**

*Histological assessment and OARSI score.*

Comparable sections from each animal, stained using haematoxylin and eosin (H&E), toludine blue and safranin O were imaged (Leica DMRB) and assessed for histopathological changes using the OARSI scoring scheme^14^ (KR blinded to genotype). The scheme assigns a score between 0 and 6 to all four quadrants averaged over three sections (n=6 of each sex and genotype at each time point): normal cartilage (0), superficial fibrillations without loss of cartilage (1), vertical clefts and loss of surface lamina (2), vertical clefts/erosion to the calcified layer with a lesion between 1-25% of the quadrant width (3), lesion covering 25-50% of the quadrant width (4), lesion covering 50-75% of quadrant width (5), lesion covering >75% of quadrant width (6). The OA severity is expressed as the summed score across the entire joint and analysed by 3 way ANOVA (SPSS). Collagen birefringence in the tibial plateau (two sections n=4 for each sex and genotype at each time point) was imaged by polarized light microscopy (Polarisation equipped Zeiss Photomicroscope).

*Immunohistochemistry*

A custom anti-NOV rabbit polyclonal antibody Novpep5170 (Pepceuticals Ltd), raised against CPQNNEAFLQDLELKTSRGEI, was used to analyse expression in 6 month *Nov^del3^+/+* and *Nov^del3^-/-* males and females (n=3 for each genotype and sex). Details of antibody staining given in supplementary methods. Antigen retrieval with 1mg/ml testicular hyaluraonidase for 30 minutes at 37˚C (Sigma) was required. Sections were incubated with 2µg/ml primary antibody in 10% goat serum overnight at 4˚C. Cell proliferation and apoptosis was analysed using a rabbit polyclonal to PCNA (Abcam) and PARP p85 (Promega), incubated at 1µg/ml in 10% goat serum for 1 hour at room temperature, following antigen retrieval with 20µg/ml Proteinase K (Sigma) (n=4 for each genotype, sex and stage). A goat anti-rabbit biotinylated secondary antibody (Vector), Vectastain elite ABC kit, DAB stain and haematoxylin counterstain were used. Sections were imaged (Leica DMRB microscope) and the percentage positive cells determined for each of four quadrants, averaged over two slides, were analysed by 3 way ANOVA (SPSS) (KR blinded to genotype).

Collagen I (ColI) and Collagen X (ColX) expression was assayed using the mouse monoclonals anti-ColI (1/1000, Sigma) and anti-ColX (1/10, kind gift of Klaus von der Mark) following retrieval with 2mg/ml pronase (Sigma) for 30 minutes at 37˚C and 1mg/ml testicular hyaluronidase (Sigma) for 1 hour at 37˚C respectively (n=4 for each genotype, sex and stage). The Vector M.O.M immunodetection kit and Nova Red substrate (Vector) were used and sections imaged (Leica DMRB microscope).
